# Mesentero-axial gastric volvulus treated with laparoscopic gastropexy: a case report

**DOI:** 10.1186/s40792-023-01596-w

**Published:** 2023-02-09

**Authors:** Masato Kawahara, Tsunehiko Maruyama, Yoshiki Kaneko, Naoaki Konno, Hiroshi Kashimura, Tatsuya Oda

**Affiliations:** 1 Department of Surgery, Mito Saiseikai General Hospital, 3-3-10, Futabadai, Mito City, Ibaraki 311-4198 Japan; 2 Department of Gastroenterology, Mito Saiseikai General Hospital, 3-3-10, Futabadai, Mito City, Ibaraki 311-4198 Japan; 3 Department of Gastroenterological Surgery, University of Tsukuba, 1-1-1, Tennodai, Tsukuba City, Ibaraki 305-8577 Japan

**Keywords:** Gastric volvulus, Mesentero-axial, Laparoscopic gastropexy, Case report

## Abstract

**Background:**

Mesentero-axial gastric volvulus (MAGV) is an uncommon subtype of gastric volvulus (GV). However, reports of such cases in adult patients are very rare. We present an unusual case of idiopathic MAGV in an old woman.

**Case presentation:**

An 84-year-old woman was referred to the emergency department for vomiting and abdominal pain. An abdominal computed tomography scan revealed a mesentero-axial gastric volvulus, which could be corrected endoscopically, and the symptoms were relieved. Contrast-enhanced examination was performed before the elective surgery to confirm the presence of short-axis dorsal 180-degree volvulus. The patient underwent laparoscopic surgery on a wait-and-watch basis. After releasing the torsion, the stomach returned to normal position. The gastric fornix was sutured to the left diaphragm and the gastric body and antrum were sutured to the abdominal wall using non-absorbable thread. Symptoms did not flare after the surgery.

**Conclusions:**

We experienced a rare case of adult MAGV presenting with incomplete obstruction. Laparoscopic gastropexy is useful when gastric decompression is achieved.

## Background

The definition of gastric volvulus is basically an acquired rotation of the stomach by 180 degrees or more, resulting in a closed loop obstruction [1]. Mesentero-axial gastric volvulus (MAGV) is an uncommon subtype of gastric volvulus (GV) that can present as a surgical emergency due to life-threatening complications [2, 3]. However, reports of such cases in adult patients are very rare. Herein, we present an unusual case of idiopathic MAGV in an old woman, with a review of the literature.

## Case presentation

An 84-year-old woman was referred to the emergency department for vomiting and abdominal pain. She had a history of similar symptoms and had been hospitalized. She was relieved with conservative treatment since a diagnosis could not be reached. She had abdominal distension, but no tenderness or muscular defenses. Her vital signs were stable. No abnormal values were found in the blood test findings.

**Fig. 1:**
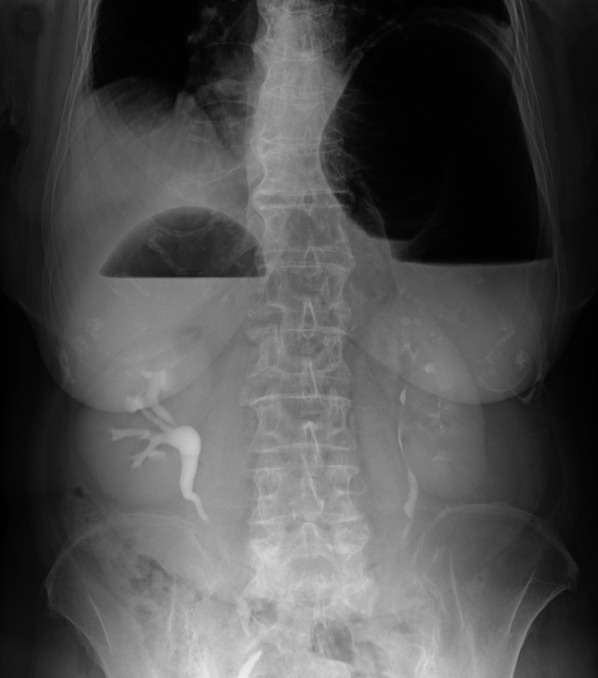
Erect chest and abdomen X-ray showed a double air-fluid level

**Fig. 2:**
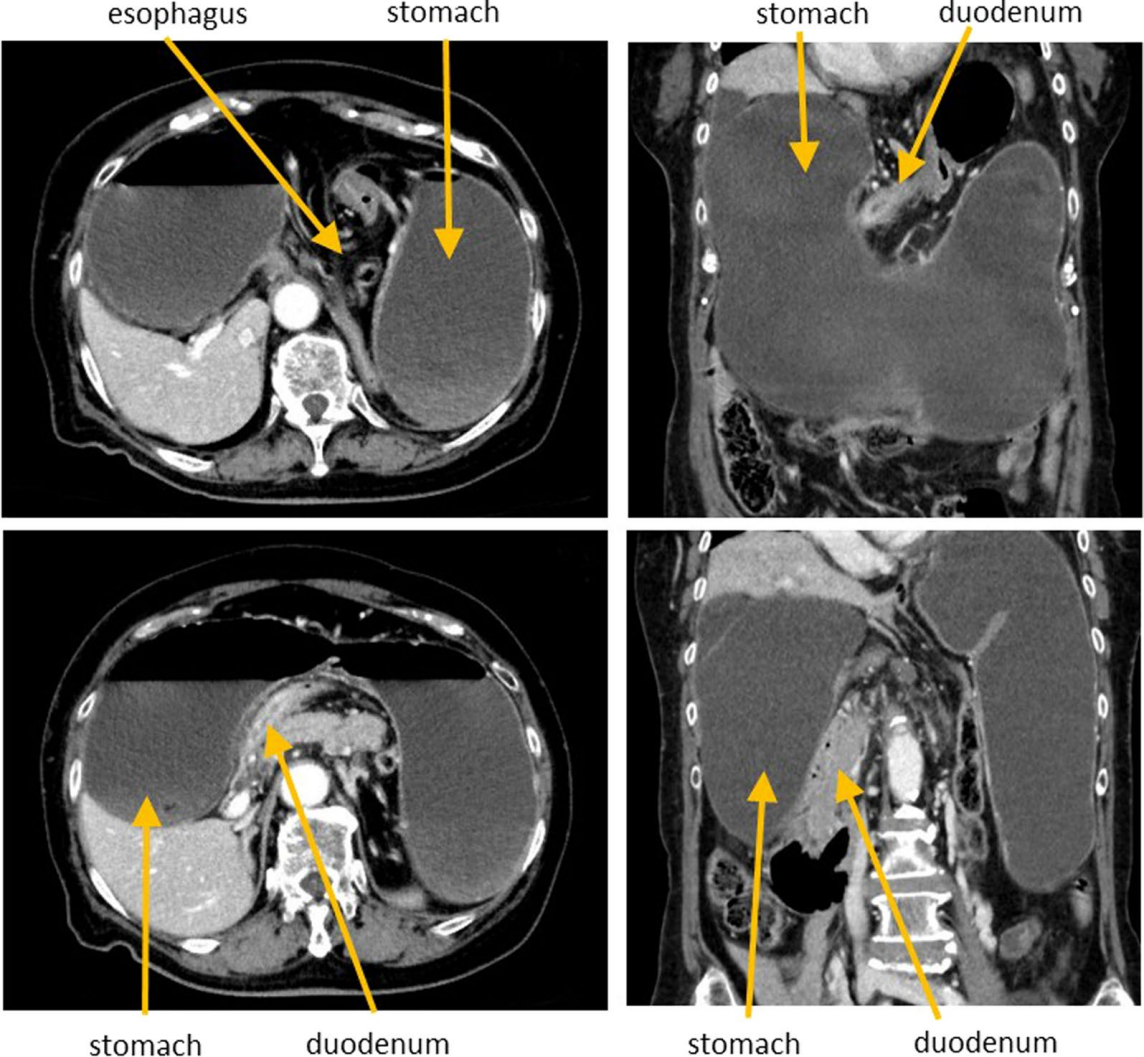
Abdominal computed tomography showed mesentero-axial gastric volvulus (MAGV) with markedly dilated stomach and stenosis of the duodenum

**Fig. 3:**
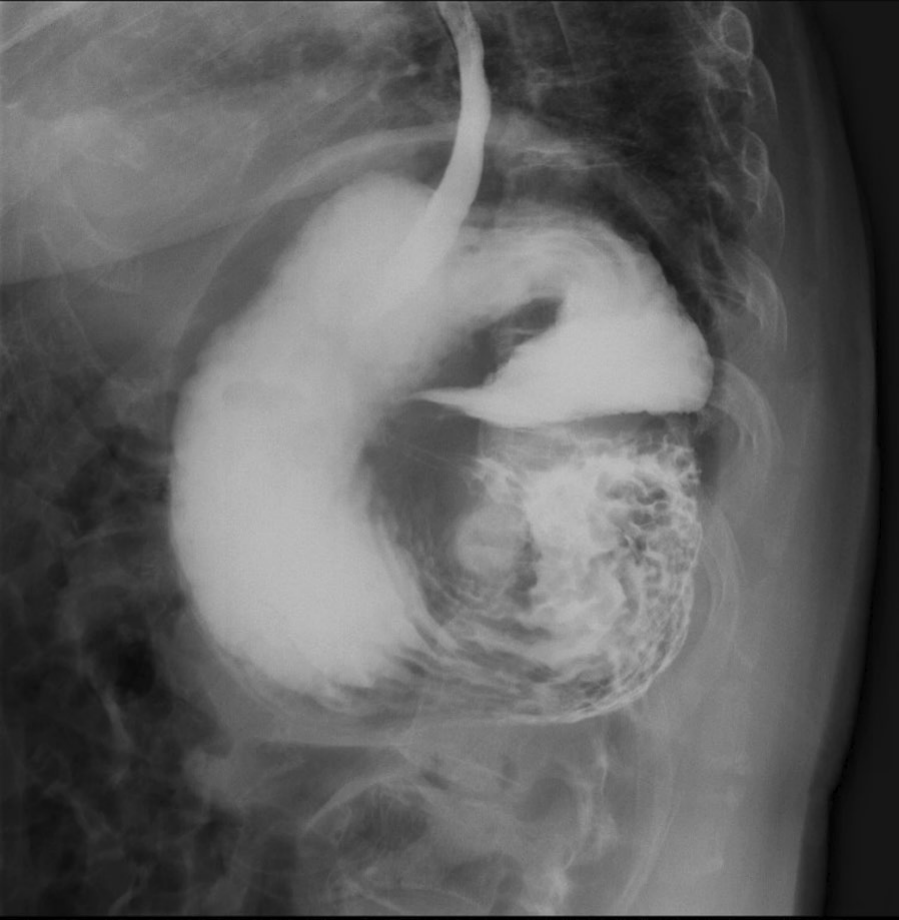
Preoperative contrast studies were performed to confirm the presence of mesentero-axis dorsal 180-degree volvulus

**Fig. 4:**
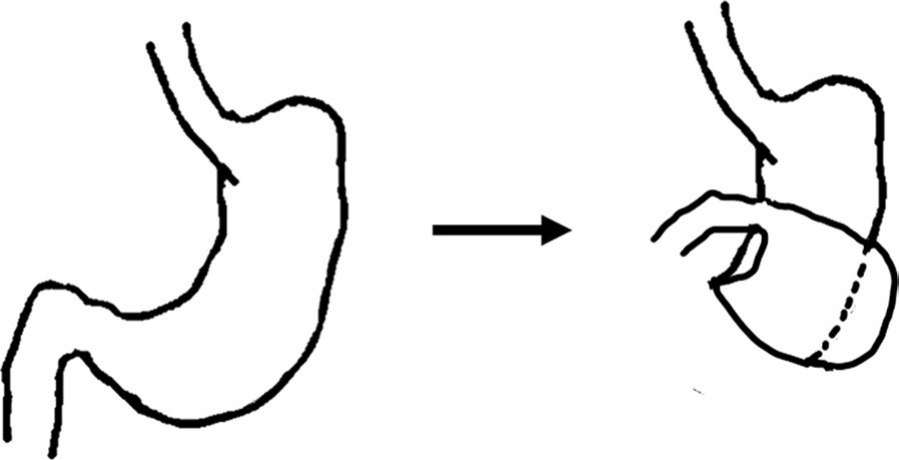
An intraoperative finding before the rotation of stomach to normal position

**Fig. 5:**
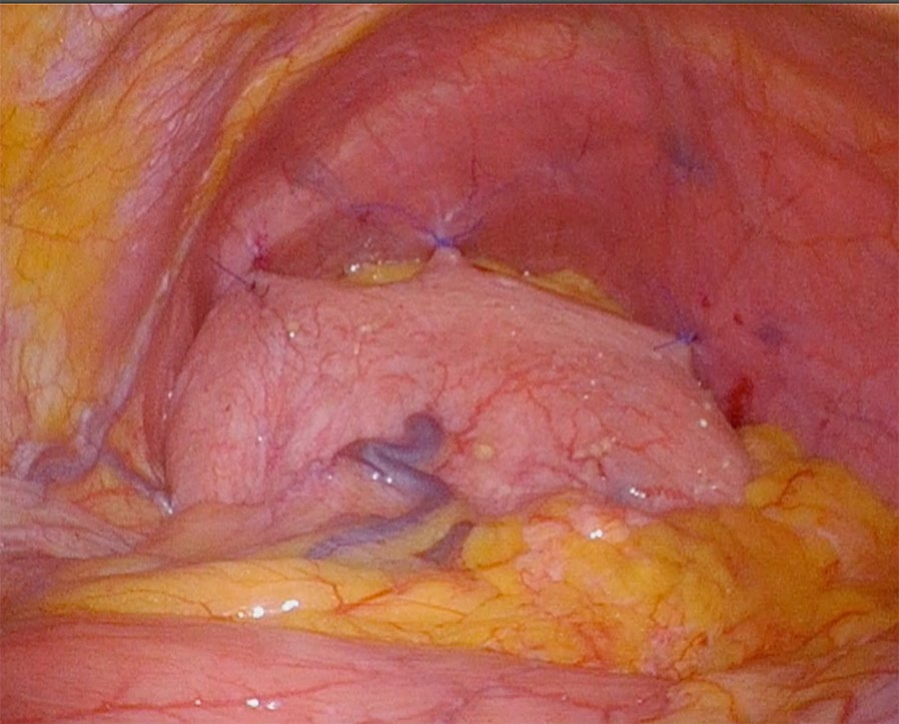
The gastric fornix was sutured to the left diaphragm

**Fig. 6:**
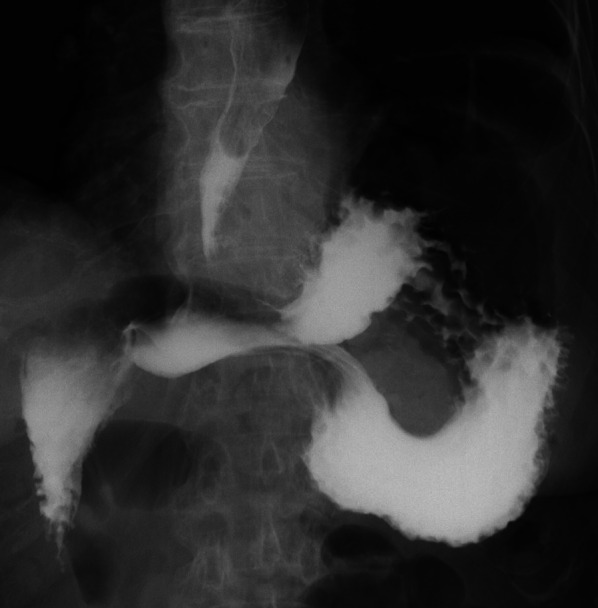
A postoperative contrast-enhanced examination showed that the stomach had returned to normal position and there was no stenosis

Erect chest and abdomen X-ray demonstrated a double air-fluid level (Fig. 1). Abdominal computed tomography (CT) identified a MAGV with the pylorus located superior to the gastro-esophageal junction (Fig. 2a–d). This pathogenesis could be corrected endoscopically, and symptoms were relieved. We diagnosed her previous symptoms as being caused by MAGV and determined that surgery was necessary for her. Contrast-enhanced examination was performed before the elective surgery to confirm the presence of mesentero-axis dorsal 180-degree volvulus (Figs. 3 and 4) and demonstrated delayed passage of contrast through the gastroduodenal junction, which was located superior to the gastro-esophageal junction in keeping with an incomplete gastric outlet obstruction. The patient underwent laparoscopic surgery on a wait-and-watch basis. After releasing the torsion, the stomach returned to normal position. The gastric fornix was sutured to the left diaphragm and the gastric body and antrum were sutured to the abdominal wall using non-absorbable thread (Fig. 5). A postoperative contrast-enhanced examination showed that the stomach had returned to normal position and there was no stenosis (Fig. 6). The patient experienced no further episodes after surgery.

## Discussion

**Fig. 7:**
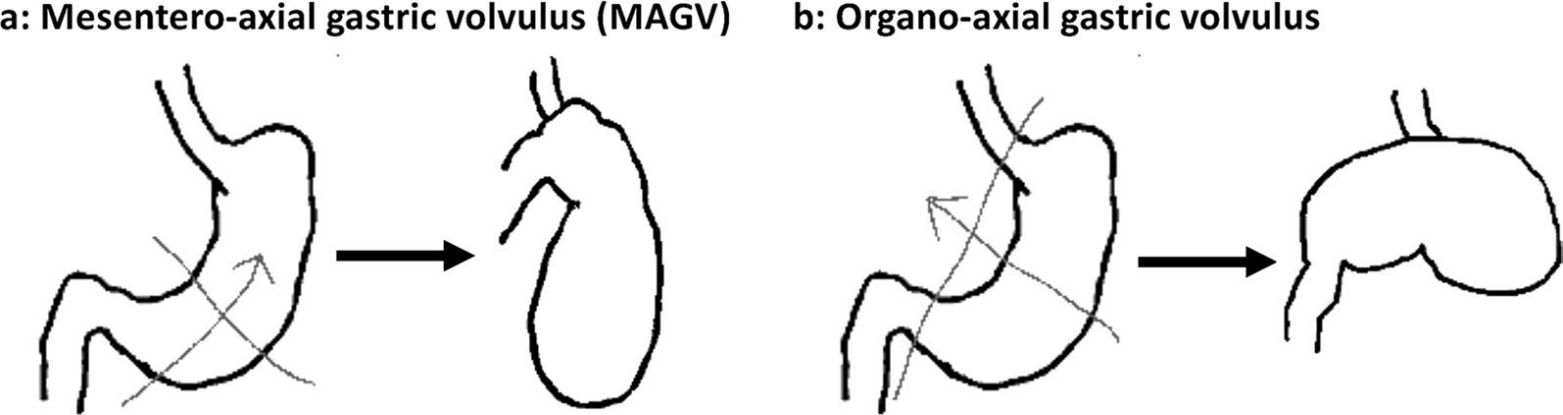
**a** Mesentero-axial GV refers to rotation of the stomach along the short axis. **b** Organo-axial GV refers to rotation along the longitudinal axis

Gastric volvulus is a rare condition occurring in both adults [2, 3] and children [4]. Gastric volvulus is defined as pathological rotation of the stomach and is classified according to the axis of gastric rotation into mesentero-axial type (59%) (Fig. 7a) and organo-axial type (29%) (Fig. 7b), and a third complex type involving rotation of both axes (12%) has also been reported [5]. MAGV is less frequent than the organo-axial subtype [5, 6] and is generally unrelated to anatomic or structural defects, whereas the organic axial subtype is generally secondary to esophageal hiatus or paraesophageal hernia.

In this case, no clear underlying cause was identified. MAGV is a rotation of the stomach around its short axis that causes the anterior wall of the stomach to fold, bringing the pylorus and antrum into close proximity to the gastro-esophageal junction [2]. Fluoroscopy helps to confirm the position of the pylorus relative to the gastro-esophageal junction and the presence and extent of obstruction.

In this case, nasogastric decompression improved symptoms, but it was decided to perform a gastropexy to prevent recurrence. The goal of surgical intervention is to fix the stomach to repair the volvulus and reduce the chance of recurrence [7]. The preferred surgical procedure is anterior gastropexy, in which the greater curvature of the stomach is fixed to the anterior abdominal wall [8]. Historically, open surgery has been the procedure of choice, but recently there have been increasing reports of laparoscopic gastropexy [9]. Although there are no randomized controlled trials, observational studies support laparoscopic surgery because of its advantages in terms of shorter hospital stay and improved postoperative morbidity. One study reported recurrence rates of 42 vs. 15% for laparoscopic and open gastropexy, respectively [10]. In laparoscopic gastropexy surgery, there are reports of single-incision surgery in pediatric patients [3]. The reason for the high recurrence rate in the old literature may be that the laparoscopic technique was still immature. In this case, we chose laparoscopic surgery because we wanted to perform minimally invasive surgery on the elderly patient. To prevent recurrence, we sutured the stomach and abdominal wall with non-absorbable thread. She was discharged from the hospital without further complications and with a short postoperative hospital stay, despite her advanced age, by laparoscopic gastropexy surgery.

## Conclusions

We present a rare case of adult MAGV presenting with incomplete obstruction. Understanding that the disease is due to this condition and that laparoscopic gastropexy is useful when gastric decompression is achieved.

## Data Availability

Applicable.

## Abbreviations

MAGV: Mesentero-axial gastric volvulus
GV: Gastric volvulus
CT: Computed tomography

## References

[1] Sevcik WE, Steiner IP (1999). Acute gastric volvulus: case report and review of the literature. CJEM.

[2] Mistry V, Gamble EL, Chang J (2020). Adult mesentero-axial gastric volvulus: case report. J Med Imaging Radiat Oncol.

[3] Takahashi T, Yamoto M, Nomura A, Ooyama K, Sekioka A, Yamada Y (2019). Single-incision laparoscopic gastropexy for mesentero-axial gastric volvulus. Surg Case Rep.

[4] da Costa KM, Saxena AK (2019). Management and outcomes of gastric volvulus in children: a systematic review. World J Pediatr.

[5] Jabbour G, Afifi I, Ellabib M, El-Menyar A, Al-Thani H (2016). Spontaneous acute mesenteroaxial gastric volvulus diagnosed by computed tomography scan in a young man. Am J Case Rep.

[6] Akhtar A, Siddiqui FS, Sheikh AAE, Sheikh AB, Perisetti A. Gastric volvulus: a rare entity case report and literature review. Cureus. 2018. https://www.cureus.com/articles/11429-gastric-volvulus-a-rare-entity-case-report-and-literature-review. Accessed 17 Sep 2022. 10.7759/cureus.2312 PMC5947932 29755908

[7] Gourgiotis S, Vougas V, Germanos S, Baratsis S (2006). Acute gastric volvulus: diagnosis and management over 10 years. Dig Surg.

[8] Wasselle JA, Norman J (1993). Acute gastric volvulus: pathogenesis, diagnosis, and treatment. Am J Gastroenterol.

[9] Al-Faraj D, Al-Haddad M, Al-Hadeedi O, Al-Subaie S (2015). A case of acute mesentero-axial gastric volvulus in a patient with a diaphragmatic hernia: experience with a laparoscopic approach. J surg case rep.

[10] Koger KE, Stone JM (1993). Laparoscopic reduction of acute gastric volvulus. Am Surg.

